# Risk factors for clinically relevant deviations in patients’ medication lists reported by patients in personal health records: a prospective cohort study in a hospital setting

**DOI:** 10.1007/s11096-022-01376-w

**Published:** 2022-01-15

**Authors:** Denise J. van der Nat, Margot Taks, Victor J. B. Huiskes, Bart J. F. van den Bemt, Hein A. W. van Onzenoort

**Affiliations:** 1 Department of Clinical Pharmacy, Breda, The Netherlands; 2 Department of Pharmacy, St. Maartenskliniek, Nijmegen, The Netherlands; 3 Department of Pharmacy, Radboud Institute for Health Sciences (RIHS), Radboud University Medical Centre, Nijmegen, The Netherlands; 4 Department of Clinical Pharmacy and Toxicology, Maastricht University Medical Center, Maastricht, The Netherlands

**Keywords:** Drug information, Medication errors, Medication reconciliation, Medication safety, Personal health record

## Abstract

*Background* Personal health records have the potential to identify medication discrepancies. Although they facilitate patient empowerment and broad implementation of medication reconciliation, more medication discrepancies are identified through medication reconciliation performed by healthcare professionals. *Aim* We aimed to identify the factors associated with the occurrence of a clinically relevant deviation in a patient’s medication list based on a personal health record (used by patients) compared to medication reconciliation performed by a healthcare professional. *Method* Three- to 14 days prior to a planned admission to the Cardiology-, Internal Medicine- or Neurology Departments, at Amphia Hospital, Breda, the Netherlands, patients were invited to update their medication file in their personal health records. At admission, medication reconciliation was performed by a pharmacy technician. Deviations were determined as differences between these medication lists. Associations between patient-, setting-, and medication-related factors, and the occurrence of a clinically relevant deviation (National Coordinating Council for Medication Error Reporting and Prevention class $\ge$ E) were analysed. *Results* Of the 488 patients approached, 155 patients were included. Twenty-four clinically relevant deviations were observed. Younger patients (adjusted odds ratio (aOR) 0.94; 95%CI:0.91–0.98), patients who used individual multi-dose packaging (aOR 14.87; 95%CI:2.02–110), and patients who used $\ge$ 8 different medications, were at highest risk for the occurrence of a clinically relevant deviation (sensitivity 0.71; specificity 0.62; area under the curve 0.64 95%CI:0.52–0.76). *Conclusion* Medication reconciliation is the preferred method to identify medication discrepancies for patients with individual multi-dose packaging, and patients who used eight or more different medications.

## Impacts on practice

- Patients using eight different medications and/or have multi-dose packaging were at greatest risk for (clinically relevant) deviations in their medication lists based on an online personal health record, compared to medication reconciliation performed by a healthcare professional.
- Our results contributed to the development of an algorithm able to calculate a risk score for deviations in personal health records based on patient characteristics.
- Patient counselling and education about how to use personal health records should be offered to patients to increase their capability to use them for medication reconciliation.

## Introduction

Transitions in healthcare impair the continuity of medication information resulting in medication discrepancies after care transitions [1–3]. Medication discrepancies are defined as inconsistencies between two or more medication lists [4]. Up to 100% of the patients admitted to the hospital have at least one medication discrepancy, of which, half of these have the potential to harm patients [5–10]. If not recognised early, medication discrepancies can lead to an increased risk of re-admissions, emergency room visits and prolonged hospital stays [11–15].

The gold-standard to identify medication discrepancies is medication reconciliation (MR) performed by a healthcare professional [16]. During MR, the best possible medication history is composed by interviewing patients and/or family, whenever possible, and by verifying and documenting medication history [16]. It is advised that all patients receive MR prior to, or within, 24 h after hospitalisation [16]. However, in practice, MR is particularly performed with patients with a greater risk of medication discrepancies, because it is a time consuming process [14, 16, 17]. Previous research indicated that different factors are associated with the number of identified medication discrepancies. In particular, the patient’s age and the number of (high-risk) medications are associated with increased medication discrepancies [10, 18–32].

Patients who use an online personal health record (PHR)—a secure online website that gives patients access to personal health information—are able to relatively accurately record a list of their medication [33, 34]. Although PHRs facilitate patient empowerment and broad implementation of MR, more medication discrepancies are identified with MR performed by a healthcare professional than with an online PHR used by patients [35]. To ensure patient safety, it must be examined which patients are best potential candidates for MR by making use of a PHR. We hypothesize that certain patient-, setting-, or medication-related factors, may be associated with deviations in the PHR compared to MR performed by a healthcare professional. Knowledge of these risk factors will give insight into how to target patients for whom MR can safely be performed through use of a PHR. Currently, only one small study (n = 13) investigated the association between patient characteristics and deviations between the medication list documented by the patient in the PHR, compared to the best possible medication history [34]. This study did not find an association between risk factors (such as age, sex, previous IT use, number of medications or pattern of use) and the number of deviations. However, limitations of this study were the number of included patients and the number of variables studied [34]. So, more insight into the effects of patient-, setting-, and medication-related factors is necessary.

### Aim

The objective of this study was to identify the factors associated with the occurrence of a clinically relevant deviation in a patient’s medication list based on a PHR (used by patients) compared to MR performed by a healthcare professional.

### Ethical approval

The study (N2019-0212) was approved by the Medical Ethics Committee of Utrecht, the Netherlands on 25–04-2019. No informed consent of patients was required, as only data of routine procedures were collected.

## Method

### Study design

A prospective cohort study was conducted at the Cardiology-, Internal Medicine- and Neurology Departments of Amphia Hospital, Breda, the Netherlands. Patients with a minimum age of 18 years-old, who were scheduled to be admitted to one of these departments during the period of March to April 2019 were eligible for this study. Three to 14-days prior to hospital admission, all patients received an invitation to verify their medication lists in the online PHR and to adjust their medication list, if necessary. At hospitalisation, MR was conducted by a pharmacy technician. Only patients with a verified medication list in the PHR and a bedside MR (at admission), or a MR by telephone (at least three-days before admission) were included. The pharmacy technician who performed MR was not informed of the medication information patients had entered into their PHRs.

### Medication reconciliation performed by a healthcare professional

At the Amphia Hospital, MR is performed by a pharmacy technician according to the standard operating procedure of the ‘High 5s project’ of the World Health Organization [16]. During this process, the best possible medication history is created by using at least two different drug information sources. Pharmacy technicians combine the information provided by a structured interview with the patients about medication use, the information from electronic health records and (if available) the information from the Nationwide Medication Record System, to obtain the best possible medication history. The Nationwide Medication Record System is a digital national network that exchanges medication dispensing data from all pharmacies in the Netherlands, provided that patients give permission to exchange this information [36, 37]. Patients were excluded from this study in cases in which no information from the Nationwide Medication Record System was available.

### Medication reconciliation performed by patients using a personal health record

For this study, we implemented a PHR (Zorgdoc®, Eindhoven, the Netherlands) specifically developed to enable patients to update their own medication list. The PHR system could be accessed with two interfaces: a website for patients, and one for healthcare professionals. Both components contained a patients’ medication file; one owned by the patients, and one by the healthcare professional. Both components were synchronized, giving the users (patients and professionals) access to the information captured in either file.

Patients received automated invitations to update their medication files approximately two-weeks prior to their visit. During the verification process, patients were asked to verify the medication information shown that was derived from the Nationwide Medication Record System. After the patient finished the verification process, a healthcare professional validated the medication information entered, and the medication list was updated in the electronical health record file.

### Outcome measures

The main outcome of the study is the patient-, setting-, and medication-related factors associated with the occurrence of a clinically relevant deviation in a patient’s medication list based on an online PHR (used by patients) compared to MR performed by a healthcare professional. A deviation was defined as a difference between the PHR and the medication list derived from MR by a pharmacy technician. The severity of the deviations were classified according to the National Coordinating Council for Medication Error Reporting and Prevention Index [38, 39]. Deviations categorised in Category E and higher were classified as clinically relevant [40]. The severity of the deviations was independently determined by two researchers (DN, MT). In case of disagreement in the severity of the deviations, a third person (HO) was consulted.

### Data collection

Based on the literature, ten potential risk factors for the occurrence of a deviation in the PHR were assessed [24, 41]. Table 1 shows the risk factors, including their sources.

**Table 1:** Collected patient-, setting-, and medication-related factors

Variable	Source of information	Additional explanation
Patient’s age	Electronic health record	–
Patient’s gender	Electronic health record	–
The number of (pre-admission) medications	Electronic health record	The number of different medications was determined from the medication list generated with medication reconciliation performed by a pharmacy technician. Both regular and ad-hoc medications were considered and combination products were counted as one medication
The number of high-risk medications	Electronic health record	Medications were classified as high-risk medications according to the Institute For Safe Medication Practices list of high-alert drugs and the Narrow Therapeutic Index list of the Royal Dutch Pharmacists’ Association [49, 50]
The number of known comorbidities	Electronic health record	The known comorbidities were extracted from the problem list of the electronic health record composed by doctors, according to the International Classification of Diseases-10. All diagnoses in the patient’s past that were reported by the doctors as ‘current’ were considered. To make sure that the list was complete and correct, the information was checked and supplemented with comorbidities based on medication information of the best possible medication history
Medical department admitted to	Electronic health record	–
Number of outpatient visits in the last twelve months	Electronic health record	Only the outpatient visits at the Amphia Hospital, the Netherlands, were considered
Use of different outpatient pharmacies in the last six months	Nationwide Medication Record System	–
The type of care before admission	Patient	–
Use of individual multi-dose packaging	Patient	In multi-dose packaging, the patients’ medication is removed from its original packaging and re-packed in disposable plastic pouches. All medication from one dosing moment is packed into a single pouch, and the pouches are labelled with the date, patient data, time of intake, and the pouches’ contents [51]

### Statistical analysis

A logistic regression analysis was performed to determine associations between the potential risk factors and the presence of a clinically relevant deviation in a patient’s medication list. A forward conditional regression analysis, in which significant risk factors (p < 0.1) were included, was performed to adjust for potential confounding. Significant continuous variables were analysed by a Receiver Operating Characteristic (ROC)-curve and the optimal cut-off point was determined with the Youden’s index. Descriptive analyses were performed to determine the number of (clinically relevant) deviations. Descriptive statistics were provided using mean (± standard deviation (SD)) or median (interquartile range [IQR] values), depending on the (non-) parametric distribution of measured variables. Results were considered statistically significant at p < 0.05. Data were analysed using IBM SPSS Statistics software, Version 25.

## Results

### Study sample

**Fig. 1:**
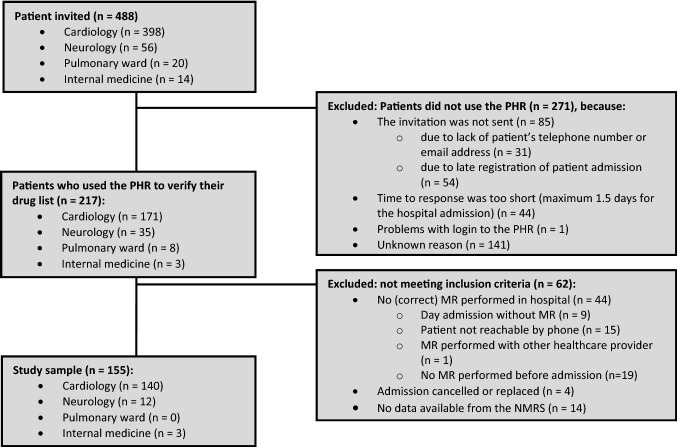
Flowchart of the study sample selection. The flowchart displays the number of patients admitted per department and the reasons for exclusion. At the end of the study, 155 patients were included. *MR* Medical reconciliation, *NMRS *Nationwide medication record system,* PHR* Personal health record

Among 488 patients initially invited, 217 (44.5%) patients completed the PHR verification, of which 155 met the inclusion criteria (Fig. 1). The main reason (71.0%) for exclusion was that MR was not performed by a pharmacy technician. Most patients (90.3%) were admitted to the Cardiology Department (Table 2). The included patients (median age 66 (IQR: 57–73) years-old, 69.0% male), used a median number of 7 (IQR: 3–10) medications, and were mostly living at home (98.7%).

**Table 2:** Patient-, setting-, and medication-related characteristics of the study sample (n = 155)

Characteristics	Study sample (n = 155)
Number of deviations^a^, median [IQR]	2.0 (1.0–3.0)
Number of clinically relevant deviations^b^, median [IQR]	0.0 (0.0–0.0)
Age (years, median (IQR))	66.0 (57.0–73.0)
Male, N (%)	107 (69.0)
Number of medications in the BPMH, median [IQR]	7.0 (3.0–10.0)
Number of high-risk medications^c^ in the BPMH, median [IQR]	0.0 (0.0–1.0)
Known comorbidities, median [IQR]	4.0 (2.0–7.0)
Number of outpatient visits in the last 12-months, median [IQR]	0.0 (0.0–1.0)
Usage of different outpatient pharmacies in the last six-months, median [IQR]	1.0 (1.0–2.0)
Living at home, N (%)	153 (98.7)
Use of individual multi-dose packaging, N (%)	5 (3.2)
Medical department admitted to, N (%)	
Cardiology Department	140 (90.3)
Neurology Department	12 (7.7)
Internal Medicine Department	3 (1.9)

*BPMH* Best possible medication history, *IQR* Interquartile range, *SD* Standard deviation

^a^Deviation was determined as a difference between the medication list composed by the patients with the PHR compared to MR performed by a pharmacy technician

^b^Deviations in Category E and higher (according to National Coordinating Council for Medication Error Reporting and Prevention) were classified as clinically relevant [40]

^c^Number of high-risk drugs according to the Institute For Safe Medication Practices high-alert medications list and the narrow therapeutic index list of the Royal Dutch Pharmacists’ Association [49, 50]

### Identified (clinically relevant) medication discrepancies in the PHR compared to MR performed by a pharmacy technician

When the PHR was directly compared to MR performed by a pharmacy technician, 37 (23.9%) patients had a medication list that was identical to the medication list of MR. Focusing on the clinically relevant deviations, 134 (86.5%) patients had a medication list that was identical. The minority (7.2%) of the deviations were clinically relevant, which corresponds to 1.4% of the total medications used.

### Risk factors for the occurrence of a clinically relevant deviation in the medication list based on a PHR compared to MR performed by a pharmacy technician

**Fig. 2:**
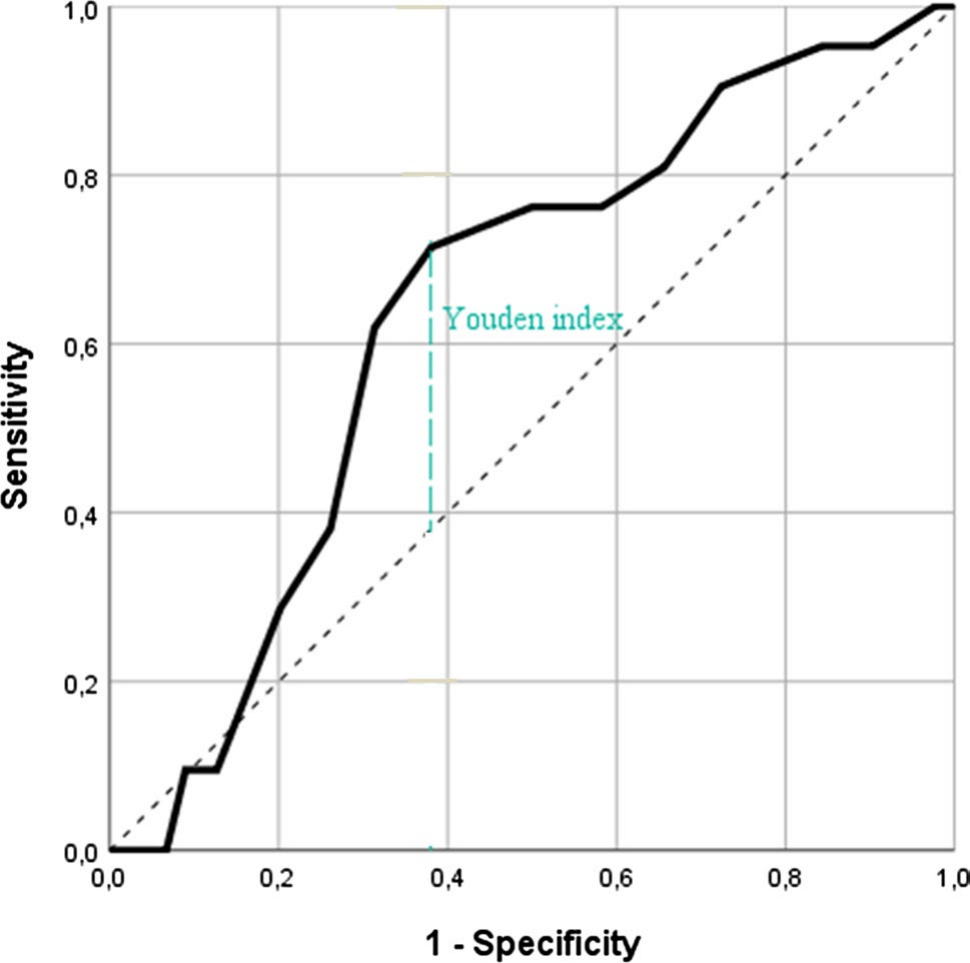
Receiver Operating Characteristic (ROC)-curve for the number of different medications for detecting a clinically relevant deviation in a patient’s medication list based on a personal health record (PHR) compared to medication reconciliation (MR). Patients with eight or more different medications were at highest risk for the occurrence of a clinically relevant deviation in their medication list based on the PHR compared to MR (Youden’s index 0.33; sensitivity 0.71; specificity 0.62; area under the curve: 0.64; 95% confidence interval: 0.52–0.76)

**Fig. 3:**
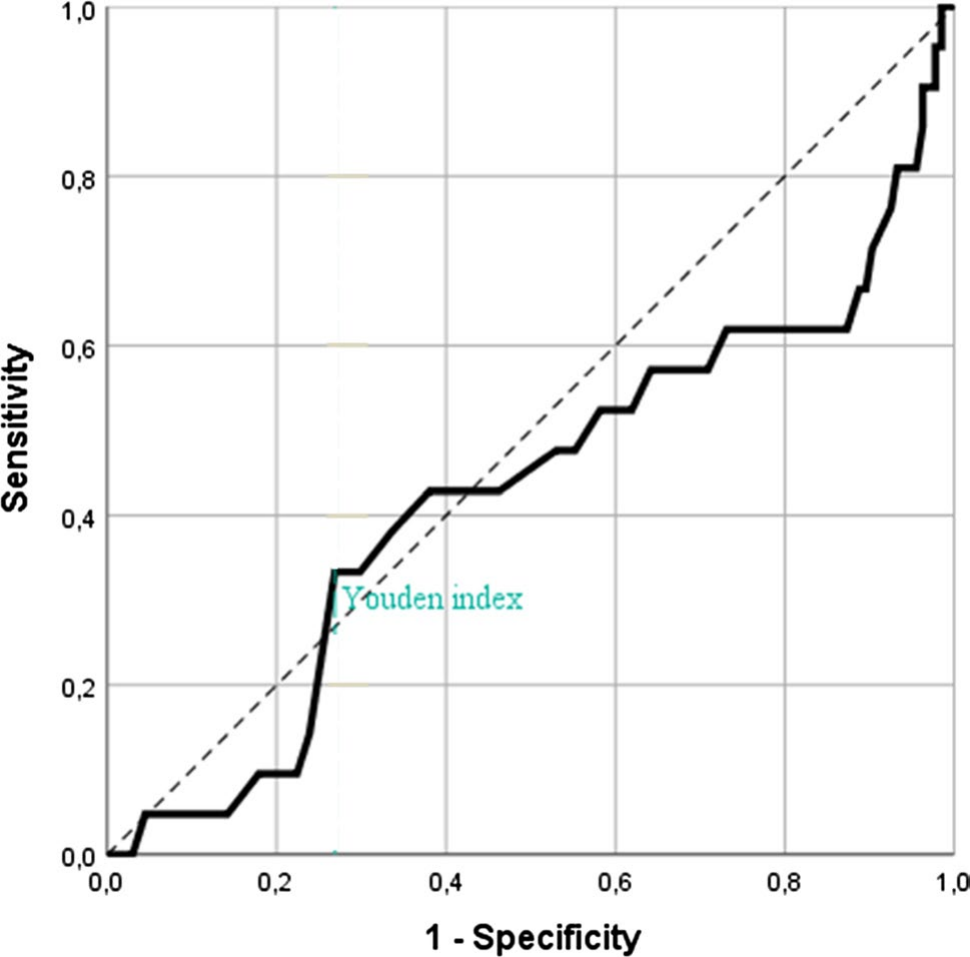
Receiver Operating Characteristic (ROC)-curve for patient’s age for detecting a clinically relevant deviation in a patient’s medication list based on a personal health record (PHR) compared to medication reconciliation (MR). Patients younger than 73 years-old were at highest risk for the occurrence of a clinically relevant deviation in their medication list based on the PHR compared to MR (Youden’s index 0.06; sensitivity 0.33; specificity 0.73; area under the curve: 0.43; 95% confidence interval 0.28–0.57)

Younger patients (adjusted odds ratio (aOR) 0.94; 95% confidence interval (95% CI) 0.91–0.98), patients who used individual multi-dose packaging (aOR 14.8; 95%CI (2.02–110) and/or patients who used a higher number of medications (aOR 1.15; 95% CI 1.01–1.32), were positively associated with the presence of a clinically relevant deviation in a patient’s medication list compared to MR performed by a pharmacy technician (Table 3). We observed that the risk for the occurrence of a clinically relevant deviation was highest when patients used eight or more different medications (sensitivity 0.71; specificity 0.62; area under the curve: 0.64 95% CI 0.52–0.76), or were younger than 73 years-old (sensitivity 0.33; specificity 0.73; area under the curve: 0.43; 95% CI 0.28–0.57) (Figs. 2 and 3).

**Table 3:** Risk factors for the occurrence of a clinically relevant deviation in patient’s medication list reported by patients in a personal health record compared to traditional medication reconciliation. Deviations in Category E and higher (according to National Coordinating Council for Medication Error Reporting and Prevention index) were classified as clinically relevant [40]

Variable	OR of a univariate analysis crude OR (95%CI)	Adjusted OR^a^ (95%CI)
Age	0.97 (0.94–1.01)**	0.94 (0.91–0.98)*
*Gender*		
Female	1.00	–
Male	0.69 (0.27–1.80)	–
Number of drugs in the BPMH	1.10 (0.98–1.23)**	1.15 (1.01–1.32)*
Number of high-risk medications^b^ in the BPMH	1.19 (0.82–1.73)	–
Number of outpatient visits in the last 12-months	0.86 (0.58–1.29)	–
Number of known comorbidities	1.04 (0.92–1.18)	–
Usage of different outpatient pharmacies in the last six-months	1.20 (0.58–2.48)	–
*Use of individual multi-dose packaging*		
No	1.00	1.00
Yes	11.00 (1.72–70.4)*	14.87 (2.02–110)*
*Living at home*		
No	1.00	–
Yes	6.65 (0.40–111)	–
*Medical department admitted to*		
Cardiology department	1.00	–
Neurology department	0.58 (0.07–4.74)	–
Internal medicine department	3.18 (0.28–36.85)	–

*BPMH* Best possible medication history; 95%CI, 95% confidence interval, *OR* Odds ratio

^a^Adjusted for patient’s age, the number of different medications and use of individual multi-dose packaging

^b^Number of high-risk medications according to the Institute For Safe Medication Practices high-alert medications list and the narrow therapeutic index list of the Royal Dutch Pharmacists Association [49, 50]

*P < 0.05

**P < 0.1

## Discussion

### Statement of key findings

In this study, we examined the association between patient-, setting-, and medication-related factors and the occurrence of a clinically relevant deviation in a patient’s medication list based on an online PHR (used by patients) compared to MR performed by a healthcare professional. We observed that patients using individual multi-dose packaging, patients younger than 73 years-old, and patients who used eight or more different medications, especially had a significantly increased risk of having a clinically relevant deviation in their medication lists based on a PHR.

### Strengths and weaknesses

Our study had several limitations. Firstly, the researchers who assessed the severity of the identified deviations were not blinded, and the classification of the severity of the deviations relied on subjective judgment of the researchers. This increased the risk of incorrectly classified clinically (ir)relevant deviations and may have biased the observed association between the risk factors and the occurrence of a clinically relevant deviation. The risk of bias was minimised by performing the assessment with two independent researchers.

Secondly, we assumed that there was no change in medication history between the time that patients entered their medications in the PHR and the time of performing MR. This so-called ‘history effect’ may have resulted in an overestimation of the occurrence of a clinically relevant deviation in the PHR compared to MR performed by a healthcare professional.

Thirdly, there may have been selection bias. Lack of experience in how to use new technological applications probably caused older patients to use a PHR less frequently [47]. As the majority of the included patients were admitted to Amphia Hospital’s Cardiology Department and the mean age of the Dutch patients admitted to Cardiology Departments is comparable to our study sample (67 versus 66 years-old), it is unlikely that selection bias occurred in our study [48]. However, other aspects that may have limited the external validity include the low participation rate of patients and the single centre character of the study.

### Interpretation

In our study, we observed that the number of medications was significantly associated with the occurrence of a clinically relevant deviation in the medication list based on an online PHR compared to MR performed by a pharmacy technician. Until now, only one study investigated the association between patient characteristics and deviations in the medication list documented by the patient in the PHR compared to the best possible medication history [34]. In contrast to our research, Marien et al. did not observe any significant association between deviations and patient-, and medication-related factors [34]. A possible explanation was that Marien et al. investigated a limited number of patient-, setting-, and medication-related factors in a small study sample (n = 13), Consequently, the external validity of this study may be low.

This is the first study to have examined the potential effect of using individual multi-dose packaging on the risk of deviations in the medication list based on an online PHR compared to MR performed by a healthcare professional. We found that using individual multi-dose packaging increased the risk of having a clinically relevant deviation in the medication list based on a PHR by 14-fold (aOR = 14.87). As only five patients used multi-dose packaging, this association requires careful interpretation. However, we anticipate that there is actually an increased risk for clinically relevant deviations for patients who use an individual multi-dose packaging, as patients receive this tool, because they are less capable of managing their own medications [42]. Consequently, they may have less knowledge about their medications in use and potentially have problems with checking the medication list in the PHR [42, 43]. Therefore, MR performed by healthcare professionals remains the preferred method to identify medication discrepancies for patients using individual multi-dose packaging.

Alongside this, high-risk patients should receive MR from a healthcare professional, and hospitals could take several actions to increase patients’ capabilities to use a PHR for MR. Firstly, patients must be educated about PHRs and the use of them. Furthermore, patient counselling should be available for patients who have problems with understanding the medication information reported in the PHR, and/or have issues or problems with use of the PHR. More patient counselling would consequently contribute to patient empowerment, which is positively associated with higher patient safety [52].

We observed that patients younger than 73 years-old were at highest risk for the occurrence of a clinically relevant deviation in the medication list based on a PHR, compared to MR performed by a pharmacy technician. This result was unexpected, as other studies found that a higher age was a predictor for medication discrepancies [18, 25, 26, 44–46]. Potential explanations were that younger patients were ‘hastier’ in verifying their medication lists, or they had more medications or medication combinations, increasing the risk of clinically relevant errors.

Although there are potential explanations why younger patients were at higher risk for the occurrence of a clinically relevant deviation, we suspect that the small, observed effect of patient’s age is not clinically relevant. The Youden’s index and the area under the ROC-curve, which were used to determine the cut off for patient’s age were low, indicating a failed model. Due to this, the cut-off value of 73 years-old should be interpreted carefully. Also, the cut-off of the number of different medications and the occurrence of a clinically relevant deviation must be carefully interpreted, as the area under the curve (0.64) indicates a poor model. As other studies also found that the number of different medications is related to the number of medication discrepancies [24, 41], we assume that the number of different medications is actually a risk factor for the occurrence of a clinically relevant deviation in the medication list based on a PHR.

## Conclusion

In conclusion, this is the first study that indicates that patients with individual multi-dose packaging and patients who use eight or more different medications are at greatest risk for having a clinically relevant deviation in their medication lists based on a PHR compared to MR performed by a healthcare professional. So, MR performed by a healthcare professional, remains the recommended procedure for these patients to identify any medication discrepancies. All other patients can safely perform MR through use of a PHR, which will contribute to better implementation of MR in hospitals. Our results, in combination with further research, may contribute to the development of an algorithm that is able to calculate a risk score based on a patient’s characteristics. After determining a cut-off value for this risk score, it may support hospitals in defining which patients MR can better receive MR performed by a healthcare professional instead of using an online PHR.
